# Silver-Coated Hip Megaprosthesis in Oncological Limb Savage Surgery

**DOI:** 10.1155/2016/9079041

**Published:** 2016-08-23

**Authors:** F. Donati, G. Di Giacomo, S. D'Adamio, A. Ziranu, S. Careri, MA. Rosa, G. Maccauro

**Affiliations:** ^1^Division of Orthopedic and Traumatology, Catholic University of the Sacred Heart, Rome, Italy; ^2^Division of Orthopedic and Traumatology, Messina University, Messina, Italy

## Abstract

Silver coating has demonstrated good antimicrobial activity and low toxicity. Silver-coated megaprostheses have been introduced in oncological musculoskeletal surgery considering the high rate of infection. We conducted a retrospective analysis on 68 cases of primary or metastatic bone tumors, affecting the proximal femur, treated between 2005 and 2016 with wide margins resection and tumor implants reconstruction. All patients were treated by the same surgeon, with antibiotic prophylaxis according to a standard protocol. In 55.9% of patients silver-coated hip hemiarthroplasty was implanted; in the remaining 44.1% uncoated megaprostheses were implanted. Patients were reevaluated recording the complications and focusing the analysis on infective complications. The average follow-up was 46.5 months. No patient has shown any sign of local or general silver toxicity. A SEM analysis was conducted on the 3-silver-coated hip hemiarthroplasty explanted confirming a severe degradation with a small amount of residual silver on the coating surface. Silver-coated hip prostheses have a lower rate of early infection than traditional implants but showed a reduction of antimicrobial activity for silver coating wear. We recommend using silver-coated prosthesis as primary implants for limb salvage surgery, in primary or metastatic bone tumors affecting the proximal femur, considering the absence of signs of toxicity and the lower rate of early infection.

## 1. Introduction

Limb salvage surgery following primary or metastatic bone tumors is the treatment of choice in young and old patients with an acceptable life expectancy. Thanks to improved surgical technique and implanted devices, prosthetic reconstruction achieves the best possible level of function in patients who need a wide resection for malignant tumor. Proximal femur is often involved in this kind of surgery with good survival and functional results but many complications are described: dislocations, deep infections, implant failures, periprosthetic fractures, and tumor relapses are among the most common possible severe complications [1, 2].

The infection rate in hip tumor hemiarthroplasty ranged from 10% to 40%, with great variability depending on the age, the resection size, and the primary malignant tumor involved. Preventing periprosthetic infection is one of the main issues, considering that when there are concomitant poor soft tissue conditions, secondary amputation is sometimes inevitable.

Oncological patients are often debilitated by tumor itself, chemotherapy, or concomitant illnesses regarding other organs and the large implants surface predisposed to bacterial colonization; for these reasons many options were proposed to prevent infections in this kind of surgery. Surely systemic treatments have a significant role and many intra- and perioperative antibiotic prophylaxes have been proposed [3, 4]. It is not easy to demonstrate a statistically significant supremacy among antibiotic prophylaxis proposed but is mandatory to choose one according infectious disease specialist indications. Among the metal with antimicrobial activity, silver has gained much interest due to its excellent antimicrobial activity coupled with low toxicity [5]. Only few case reports showed local sign of toxicity, like dermal argyria [6]. Silver-coated hip megaprostheses have been introduced in medical practice almost 25 years ago initially with the aim of treating local periprosthetic infections and have been recently proposed as first implant in patients with an acceptable life expectancy, in order to prevent the infections onset [7].

**Figure 1:**
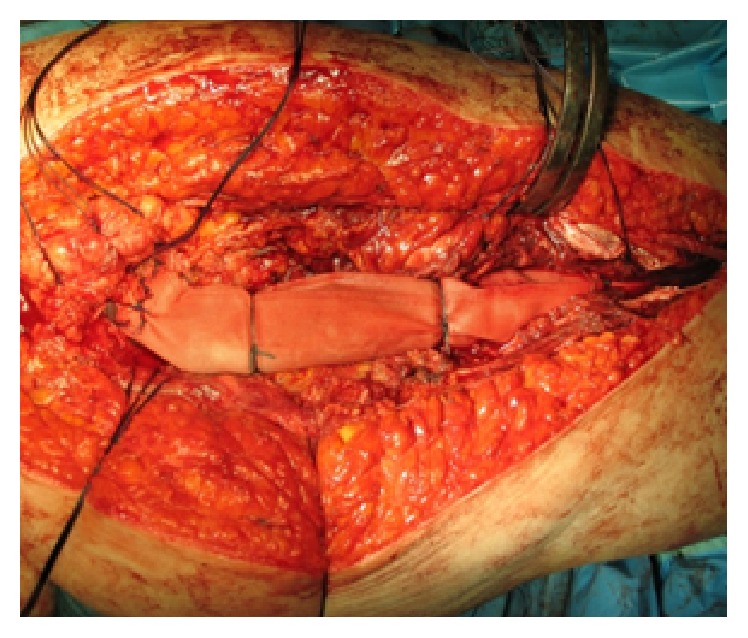
Soft tissue reconstruction using Trevira Tube after proximal femur resection and silver-coated implant.

In the Division of Orthopedic and Traumatology of “A. Gemelli Hospital” (Catholic University of the Sacred Heart, Rome) the use of silver-coated hip megaprosthesis as first implant in this kind of surgery began, in selected case almost 15 years ago, and involved in the last years more and more patients showing good functional and survival results. To improve functional results and faster recovery, reducing the dislocation rate, our surgical technique involves the use of Trevira Tube® in order to guarantee soft tissue and capsular reconstruction as described by other authors [8] (Figure 1).

Purpose of the study is to evaluate the results of silver-coated hip hemiarthroplasty compared to patients treated with uncoated hip megaprosthesis.

## 2. Materials and Methods

A retrospective study was performed, analyzing all patients affected by primary or metastatic proximal femur tumors, treated with wide margins resection and megaprostheses reconstruction by the same surgeon between 2005 and 2016 in our Department with a minimum of 12-month follow-up. All patients received the same antibiotic prophylaxis consisting in 2 mg of Cefazolin administered 30 minute before surgery followed by 1 mg each 12 hours in the three postoperative days. The main surgeon considered the implantation of a silver-coated prosthesis when technically available and when not contraindicated according to infectious disease specialist evaluation, considering the high risk of infection for this type of surgery. In other cases standard titan megaprostheses were implanted.

All the patients were periodically evaluated in our outpatients clinic recording complications and functional outcomes. Infection was diagnosed with a clinical evaluation showing a sinus tract communicating with the prosthesis or in presence of purulence in the affected joint or in case of bacterial isolation and identification from at least two separate tissue or fluid sample obtained from the affected prosthetic joint. Elevated CRP and ESR were used as marker of infection if associated with specific clinical signs. When necessary, the patients undergone antibiotic treatment based on microbiological exams and following the indication of an expert infectious disease doctor. The cases managed only with conservative treatments were defined as superficial or transitory infections and were not included in this analysis considering the high rate of such short antibiotic treatment and the objective difficulties in obtaining trustable information in a retrospective study protocol. The patients not responsive to antibiotic treatment were considered affected by a severe deep infection and required surgical revision. We differentially analyzed early infections (defined as an infection that required a second surgery before 6 months after the first surgery) and late infections.

In the data analysis, we considered the data recorded in the last follow-up available. The primary objective of the study was to compare the infection rate in the group in which hip silver-coated prostheses were implanted versus the group in which the standard titan megaprostheses were implanted. The two groups were homogenous for gender, age, resection size, time of infection, associated therapy (radio/chemotherapy or other surgeries), second surgery for other complications, and use of Trevira Tube.

Assuming a reduction of silver activity, a macroscopic visual analysis (MVA) and a scanning electron microscopy (SEM) analysis on the explanted silver-coated prostheses were performed to detect the degradation level of the silver coating. The macroscopic visual analyses were performed by 3 different authors that classified the level of degradation in 4 groups (1: no degradation, 2: initial degradation, 3: advanced degradation, and 4: coating absence) and measured the percentage of the prosthetic surface involved in degradation processes.

After macroscopic analyses, selected sections were cut following a standard laboratory procedure designed to avoid surface damages during preparation and then analyzed by field emission gun scanning electron microscopy (FEG-SEM) (LEO 1520, Oberkochen, Germany) with backscatter Centaurus detector (KE Developments, Cambridge, UK). Grain size was measured by SEM-coupled image analysis using the linear intercept method.

Close clinical surveillance was observed at each follow-up to monitor the risk of local or general silver toxicity.

## 3. Results

The overall population counted 68 patients, treated with limb salvage surgery: 31 males and 37 females. The average age was 61.6 years (range 21–78 years). In 23 cases the disease for which the patients had been treated was a primary bone tumor; in the remaining 45 cases it was secondary to a metastatic disease. The primary bone tumors were in 9 patients osteosarcoma; 7 Ewing sarcoma/primitive neuroectodermal tumor; 4 chondrosarcoma; 2 malignant fibrous histiocytoma of the bone; 1 locally advanced stage III giant cell tumor of the bone. Seven patients had pathologic fractures.

**Figure 2:**
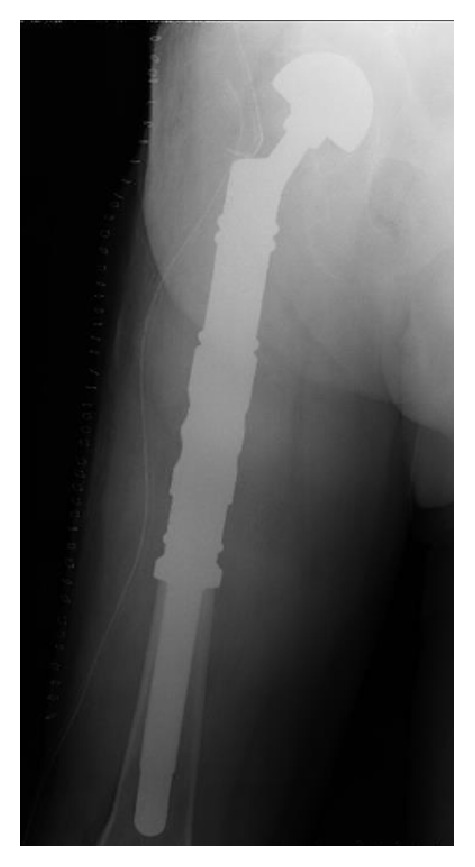
Postoperative X-ray after proximal femur resection and reconstruction with silver-coated modular prosthesis (MUTARS Implantcast Ltd., Buxtehude, Germany).

In 38 cases (55.9% of patients) silver-coated modular hip hemiprostheses (MUTARS® Implantcast Ltd., Buxtehude, Germany) were implanted (Figure 2); in the remaining part (30 cases, 44.1%), uncoated titan modular tumor hip prosthesis (MUTARS Implantcast Ltd., Buxtehude, Germany) was implanted.

The two groups were homogeneous for all the considered characteristics (Table 1). The dimension range of the bone resection was between 12 cm and 28 cm (average 16.7 cm). All patients underwent radio and/or chemotherapy when indicated.

The average follow-up was 46.5 months (range 12–114 months). 19.2% of patients died on average 35.3 months after operation. The case of death was equally distributed in silver-coated and silver-uncoated group.

Complications that required performing a second surgery were recorded in 14 cases (20.6% of the population); 2 local relapses, 4 endoprosthesis dislocations, and 8 infections (11.8%) were registered.

The overall rate of infection was 11.8%, in onsets at an average time of 25 months after first surgery. The infection rate in silver-coated prosthesis was 7.9% (3 cases). In the uncoated prosthesis group, the infection rate was 16.7%. Considering early infection cases silver-coated prosthesis group showed 1 case of infection (2.6%) versus 3 cases verified in the control group (10%). The difference between the two groups was not relevant for late infection rate (5.3% versus 6.6%) (Table 2). The differences between the two groups were not statistically significant for the small number of cases.

MVA and SEM analyses were carried out on the 3 silver-coated prostheses explanted. The two proximal femur megaprostheses affected by late infection were explanted 27 months and 18 months after surgery. In both cases an important degradation of the coating surface compared with the silver-coated megaprosthesis explanted for early infection was confirmed (4 months from first implantation).

**Figure 3:**
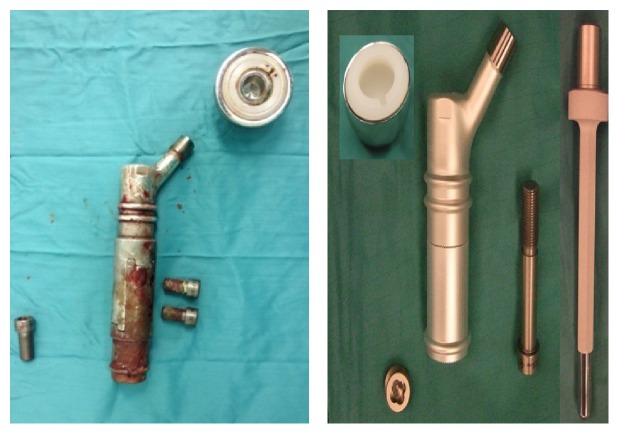
Silver-coated proximal femur prosthesis explanted 27 months after surgery compared with a new silver-coated prosthesis.

The MVA for the first case (27 months post-op) showed a grade 4 degradation for <30% of the prosthesis surface and a grade 3 degradation for >50% of prosthesis surface (Figure 3).

The second case (18 months post-op) showed a grade 3 degradation for 50% of the prosthesis surface and a grade 2 degradation for 25% of prosthesis surface.

The third case (4 months post-op) had a grade 2 degradation for less than 30% of his surface.

**Figure 4:**
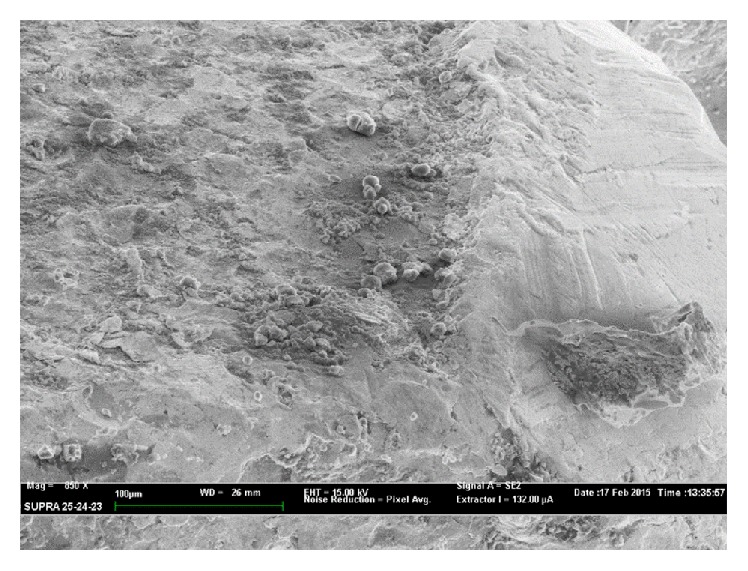
SEM analysis in a silver-coated proximal femur endoprosthesis explanted 18 months after surgery showed evident sign of wear; few, small silver grains were found.

**Figure 5:**
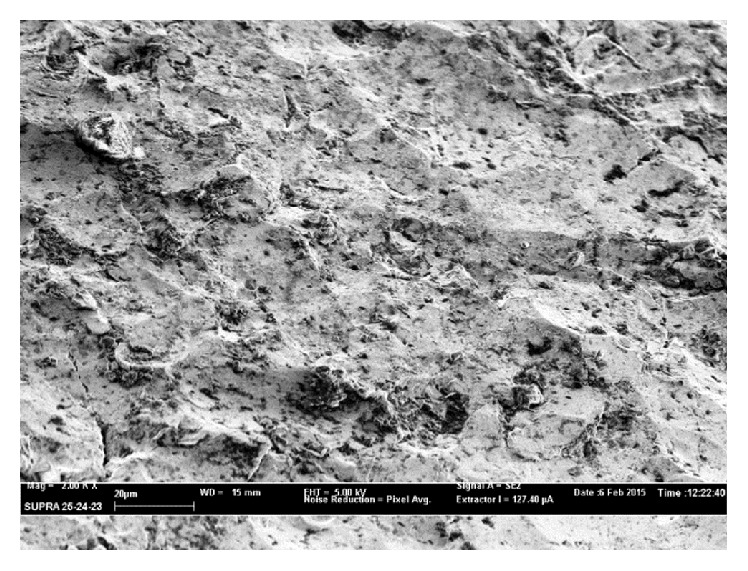
SEM analysis in a silver-coated proximal femur endoprosthesis explanted 27 months after surgery. Coating wear appears more evident: silver particles were almost disappeared.

SEM analyses confirmed severe disruption of prosthetic surface in both explanted prostheses for late infection. In the second case (Figure 4) few, small silver grains on the surface were present which were not found in the first case analyzed (Figure 5) where silver was almost completely absent.

**Figure 6:**
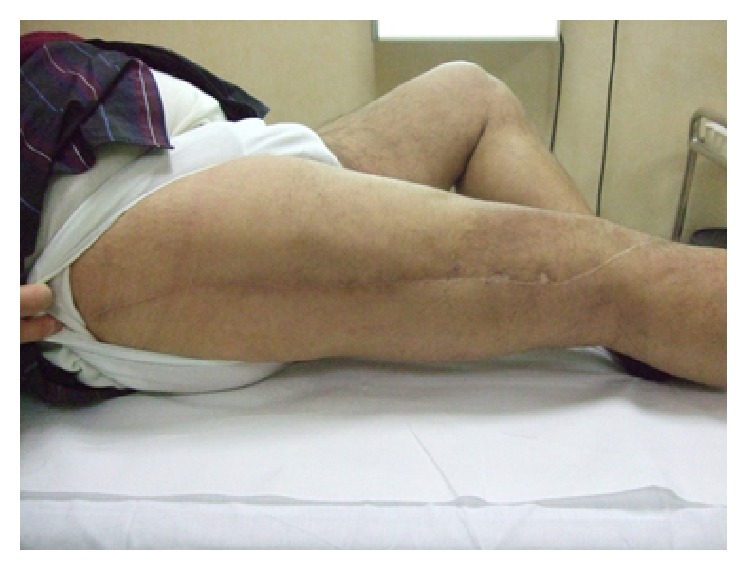
Long term clinical follow-up did not show general or local sign of silver toxicity, even in wider resection and at each time considered.

No difference between silver-coated prosthesis and uncoated tumor prosthesis group was recorded for functional scores considered. No patient ever showed local or general sign of toxicity secondary to silver exposition at each time considered, even in wider resection (Figure 6).

## 4. Discussion

Life expectancy of oncologic patients with bone metastases has remarkably increased over recent years; this has led to a higher risk of pathologic fractures and to an increased incidence of limb salvage surgery procedures [9]. Periprosthetic infection in this kind of surgery is still a common and major complication in orthopedic oncology.

It is currently impossible avoiding completely periprosthetic infections, despite the use of operating rooms with laminar airflow, systemic antibiotic treatment, and routine screening for multidrug-resistant bacteria that are becoming more and more common causes of infection. The production of an effective zone of inhibition by an antimicrobial silver-coated surface may be useful to prevent the adherence of organisms (in a manner that allows leaching of silver off the coated surface) [10, 11], not only to the coated surface but also to a variety of host-derived adhesins, such as fibronectin, fibrinogen, fibrin, and laminin that exist within the biofilm layer [12, 13].

Many authors have previously demonstrated that the silver coating of a prosthetic implant can decrease the reinfection rate, due to the release of silver ions, which produces a zone of inhibition, and the resulting coated prostheses are more likely to be infection-resistant in vivo [14–16].

The present study confirmed the protective role of silver coating compared with standard titan megaprosthesis, especially in the first 6 months after surgery. We observed that the silver coating has partially lost his full effect by the time, due to his physiological mechanical erosion. It is currently unknown what factors influence the silver coating wear in vivo, but in our experience the residual silver, present on prosthetic surface 27 months after his implant, is apparently no more sufficient in producing an effectiveness antimicrobial activity.

The most important bactericidal mechanism of the silver ions is the interaction with the thiol groups of the L-cysteine residue of proteins and its inactivation of bacterial enzymatic functions [17, 18]. Another antimicrobial mechanism is the release of potassium [19], bonding to DNA [20], and generation of intracellular reactive oxygen species (ROS). All this activity is correlated with silver ions density released by prosthetic surface. Considering the demonstrated severe degradation of the coating surface, with almost complete absence of silver, a drastic reduction of free silver ions around prosthesis is predictable. It could explain why infection risk between silver-coated prostheses and titan one is comparable for late infection.

It remains unclear if silver coating may lead to other characteristics of infection (e.g., time of infection and virulence) or to more frequent soft tissue infections. In literature, leukocyte scintigraphy has demonstrated an increased uptake, particularly in the superficial soft tissues. In our view, this may be explained by the fact that active free silver ions bind to proteins and become inactivated (silver ions may build complexes with serum albumin) [16]. In these areas, the silver coating is unable to develop an adjuvant effect as demonstrated by Schierholz et al. [17]: free silver ions may precipitate in albumin-containing environments (e.g., hematoma), leading to concentrations that are too low for bactericidal effects to be achieved. In our study rate of infection was not significantly related to this kind of complication or to the dimension of the resection, probably because of the small number considered, but we feel free to confirm the suggestion that surgeon has to avoid hematoma and poor muscle coverage of the prosthesis, resulting in superficial wound healing problems, which can cause a bacterial colonization. For this reason we routinely use from one to three wound drainages up to 2 days after surgery.

Hussmann et al. [21] analyzed mass spectrometry of the wound fluid of the Redon bottles, at 7th and 14th days postoperatively to find silver concentration and CRP level. Patients with a relatively large amount of released silver ions showed a faster decrease in the inflammatory marker CRP, even if it is unclear whether the concentration of released silver ions in the immediate surroundings of the prosthesis has an influence on the clinical course.

In comparison to other metals with antimicrobial activity (like copper, cadmium, and mercury) [5], silver has shown good antimicrobial activity and low toxicity. Silver toxicity has been reported to occur at serum levels as low as 0.3 mg/mL and manifests as argyria, leukopenia, and alterations in renal, hepatic, and neural tissues. Thus, it is prudent to incorporate silver onto the surfaces of the prostheses in concentrations that are adequate to reduce bacterial adherence but not high enough to cause systemic toxicity [22, 23]. Different kinds of silver coatings have been developed and introduced for clinical practice, following accurate in vitro studies and animal testing that showed a tolerable release of silver ions and no problem in prosthetic osseous integration [14, 24]. We must underline the fact that different industrial procedures used for producing different kinds of silver coatings would probably have a different kinematics in silver ions release. Comparative studies regarding possible differences in local and general silver ions concentration and consequently different efficacy and timing of coating wear in vivo have never been performed. However no general side effects in silver-coated megaprostheses implanted in humans have ever been demonstrated for all the different silver coatings tested, with silver blood level well beyond the threshold level of toxicity [6, 21]. Few cases of local argyria have been described especially as cutaneous manifestation apparently not related to blood, urine, or aspiration fluids silver levels and without concomitant signs of renal, liver, or neurologic sufferings, concluding that the short-term surveillance of blood silver levels in these patients is not required [25, 26].

In the present study and in our previous experiences [7], no patient had ever shown any significant local or general sign of toxicity secondary to silver ions exposition.

At last, the economic factor has to be considered. Silver-coated megaprostheses are admittedly 5–7% more expensive than the other tumor prostheses [14], but considering the significant decrease in the period of hospitalization and in revision surgeries, following an overall lower infection rate, we actually adopt silver-coated prostheses as first choice implant in all primary and revision limb salvage surgery procedure in oncological patients.

## 5. Conclusions

Silver-coated hip megaprostheses are safe and useful to improve the clinical outcome in limb salvage surgery, considering the lack of toxicological signs and the lower rate of early infections. Therefore, we recommend to use silver-coated hip hemiarthroplasty as primary implants in all the primary or metastatic bone tumors involving the proximal femur.

The analyses performed on the explanted prostheses suggest a reduced antimicrobial activity of the silver coating after 6–18 months from prosthetic implantation, probably due to his degradation. In the future more studies are necessary to confirm these results and to analyze and to compare the in vivo action of different silver-coating manufactures.

## Figures and Tables

**Table 1:** Main characteristics of the analyzed population. The two groups were homogeneous for the considered parameter.

	Uncoated prosthesis	Silver-coated prosthesis	Overall population
Number of patients (male : female)	30 (14 M : 16 F)	38 (17 M : 21 F)	68 (31 M : 37 F)
Age at first surgery	60.1 y (23–75 y)	62.8 y (21–78 y)	61.6 y (21–78 y)
Primary bone tumor (PBT) : metastatic lesion (ML)	9 PBT : 21 ML	14 PBT : 24 ML	23 PBT : 45 ML
Bone resection size (cm)	14.7 cm (12–22 cm)	18.3 cm (12–28 cm)	16.7 cm (12–28 cm)
Use of Trevira Tube	93.3%	94.7%	94.2%
Follow-up (months)	51.2 (12–114 months)	42.8 (12–97 months)	46.5 (12–114 months)
Death (time of death)	20.0% (34.7 months)	18.4% (35.8 months)	19.2% (35.3 months)
Complications requiring surgery	7 (23.3%)	7 (18.4%)	14 (20.6%)

**Table 2:** Early infection, considered as evidence of infection which occurred before 6 months after first surgery, was lower in silver-coated prosthesis group. No difference was demonstrated between the two groups for late infection risk.

	Early infections	Late infections	Total
Silver-coated hip megaprostheses	1 (2.6%)	2 (5.3%)	3 (7.9%)

Titan uncoated hip megaprostheses	3 (10%)	2 (6.6%)	5 (16.7%)

## References

[1] Palumbo B. T., Henderson E. R., Groundland J. S. (2011). Advances in segmental endoprosthetic reconstruction for extremity tumors: a review of contemporary designs and techniques. *Cancer Control*.

[2] Puchner S. E., Kutscha-Lissberg P., Kaider A. (2015). Outcome after reconstruction of the proximal tibia—complications and competing risk analysis. *PLoS ONE*.

[3] Racano A., Pazionis T., Farrokhyar F., Deheshi B., Ghert M. (2013). High infection rate outcomes in long-bone tumor surgery with endoprosthetic reconstruction in adults: a systematic review. *Clinical Orthopaedics and Related Research*.

[4] Hardes J., Gebert C., Schwappach A. (2006). Characteristics and outcome of infections associated with tumor endoprostheses. *Archives of Orthopaedic and Trauma Surgery*.

[5] Tobin E. J., Bambauer R. (2003). Silver coating of dialysis catheters to reduce bacterial colonization and infection. *Therapeutic Apheresis and Dialysis*.

[6] Hardes J., Ahrens H., Gebert C. (2007). Lack of toxicological side-effects in silver-coated megaprostheses in humans. *Biomaterials*.

[7] Donati F., Di Giacomo G., Ziranu A. (2015). Silver coated prosthesis in oncological limb salvage surgery reduce the infection rate. *Journal of Biological Regulators & Homeostatic Agents*.

[8] Gosheger G., Hillmann A., Lindner N. (2001). Soft tissue reconstruction of megaprostheses using a trevira tube. *Clinical Orthopaedics and Related Research*.

[9] von Eiff C., Peters G., Heilmann C. (2002). Pathogenesis of infections due to coagulase-negative staphylococci. *The Lancet Infectious Diseases*.

[10] Sheehan E., McKenna J., Mulhall K. J., Marks P., McCormack D. (2004). Adhesion of *Staphylococcus* to orthopaedic metals, an in vivo study. *Journal of Orthopaedic Research*.

[11] Herrmann M., Vaudaux P. E., Pittet D. (1988). Fibronectin, fibrinogen, and laminin act as mediators of adherence of clinical staphylococcal isolates to foreign material. *The Journal of Infectious Diseases*.

[12] Vaudaux P., Pittet D., Haeberli A. (1989). Host factors selectively increase staphylococcal adherence on inserted catheters: a role for fibronectin and fibrinogen or fibrin. *Journal of Infectious Diseases*.

[13] Ahrens H., Gosheger G., Streitbürger A., Gebert C., Hardes J. (2006). Antimikrobielle Silberbeschichtung von Tumorprothesen. *Der Onkologe*.

[14] Gosheger G., Hardes J., Ahrens H. (2004). Silver-coated megaendoprostheses in a rabbit model—an analysis of the infection rate and toxicological side effects. *Biomaterials*.

[15] Hardes J., von Eiff C., Streitbuerger A. (2010). Reduction of periprosthetic infection with silver-coated megaprostheses in patients with bone sarcoma. *Journal of Surgical Oncology*.

[16] Shahabadi N., Maghsudi M., Ahmadipour Z. (2012). Study on the interaction of silver(I) complex with bovine serum albumin by spectroscopic techniques. *Spectrochimica Acta Part A: Molecular and Biomolecular Spectroscopy*.

[17] Schierholz J. M., Lucas L. J., Rump A., Pulverer G. (1998). Efficacy of silver-coated medical devices. *Journal of Hospital Infection*.

[18] Kim T. N., Feng Q. L., Kim J. O. (1998). Antimicrobial effects of metal ions (Ag+, Cu2+, Zn2+) in hydroxyapatite. *Journal of Materials Science: Materials in Medicine*.

[19] Tweden K. S., Cameron J. D., Razzouk A. J., Holmberg W. R., Kelly S. J. (1997). Biocompatibility of silver-modified polyester for antimicrobial protection of prosthetic valves. *Journal of Heart Valve Disease*.

[20] Wan A. T., Conyers R. A. J., Coombs C. J., Masterton J. P. (1991). Determination of silver in blood, urine, and tissues of volunteers and burn patients. *Clinical Chemistry*.

[21] Hussmann B., Johann I., Kauther M. D., Landgraeber S., Jäger M., Lendemans S. (2013). Measurement of the silver ion concentration in wound fluids after implantation of silver-coated megaprostheses: correlation with the clinical outcome. *BioMed Research International*.

[22] von Eiff C., Proctor R. A., Peters G. (2001). Coagulase-negative staphylococci: pathogens have major role in nosocomial infections. *Postgraduate Medicine*.

[23] Herrmann M., Peters G., Seifert H., Jansen B., Farr B. M. (1997). Catheter-associated infections caused by coagulase-negative staphylococci: clinical and biological aspects. *Catheter-Related Infections*.

[24] Hauschild G., Hardes J., Gosheger G. (2015). Evaluation of osseous integration of PVD-silver-coated hip prostheses in a canine model. *BioMed Research International*.

[25] Karakasli A., Hapa O., Akdeniz O., Havitcioğlu H. (2014). Dermal argyria: cutaneous manifestation of a megaprosthesis for distal femoral osteosarcoma. *Indian Journal of Orthopaedics*.

[26] Glehr M., Leithner A., Friesenbichler J. (2013). Argyria following the use of silver-coated megaprostheses: no association between the development of local argyria and elevated silver levels. *Bone and Joint Journal*.

